# Microbiological and Clinical Characteristics of Pediatric Sepsis Patients with and without Septic Shock: A Retrospective Study at a Tertiary Pediatric Hospital in China

**DOI:** 10.3390/children12091146

**Published:** 2025-08-28

**Authors:** Kai-Cheng Peng, Qin-Yuan Li, Lin Chen, Yan Zhao, Hui Liu, Zhen-Xuan Kong, Zheng-Xiu Luo

**Affiliations:** Department of Respiratory Children’s Hospital of Chongqing Medical University, National Clinical Research Center for Child Health and Disorders, Ministry of Education Key Laboratory of Child Development and Disorders, Chongqing Key Laboratory of Child Rare Diseases in Infection and Immunity, Key Laboratory of Children’s Vital Organ Development and Disease of Chongqing Health Commission, Chongqing 400014, China; 2022140140@stu.cqmu.edu.cn (K.-C.P.); liqinyuan@hospital.cqmu.edu.cn (Q.-Y.L.); 2022140139@stu.cqmu.edu.cn (L.C.); zhao.yan@hospital.cqmu.edu.cn (Y.Z.); 2023130278@stu.cqmu.edu.cn (H.L.); 2022110405@stu.cqmu.edu.cn (Z.-X.K.)

**Keywords:** sepsis, septic shock, pathogens, risk factors

## Abstract

**Highlights:**

**What are the main findings?**

- Pediatric septic shock patients exhibited significantly higher mortality (17.4% vs. 4.7%, *p* < 0.001) and prolonged hospitalization (14.8 vs. 12.0 days, *p* = 0.003) compared to non-septic-shock sepsis patients.
- Elevated lactate (OR = 1.49, 95% CI = 1.29–1.75) and pulmonary infection (OR = 2.25, 95% CI = 1.35–3.73) were identified as independent risk factors for septic shock progression.

**What is the implication of the main finding?**

- Early monitoring of lactate levels and pulmonary infection status could improve risk stratification for septic shock in pediatric sepsis, enabling timely interventions.
- Antibiotic stewardship optimization is critical, as combination antibiotic therapy was frequently used in viral (79%) and fungal (86.5%) sepsis despite limited efficacy, prolonging hospitalization in fungal cases (20.74 vs. 12.97 days, *p* = 0.017).

**Abstract:**

Background: Pediatric sepsis, a life-threatening condition, often progresses to septic shock. However, microbiological and clinical profiles between pediatric sepsis patients with and without septic shock remain underexplored. Methods: This retrospective cohort study included 1200 pediatric sepsis patients (Phoenix Sepsis Score ≥ 2) from the Children’s Hospital of Chongqing Medical University between June 2018 and June 2023. Pediatric sepsis patients with septic shock were diagnosed based on the Phoenix Cardiovascular Score being ≥1. Clinical data and pathogens were taken from the electronic medical records and analyzed. Univariate and multivariable logistic regressions were conducted to identify the risk factors for septic shock. Results: Septic shock patients had longer hospital stays (14.8 vs. 12.0 days, *p* = 0.003) and higher mortality (17.4% vs. 4.7%, *p* < 0.001) when compared to non-septic-shock patients. Among these two groups, the pathogen prevalence revealed that bacterial pathogens dominated (48.9%), followed by viruses (10.3%). Acinetobacter baumannii, Escherichia coli, and Staphylococcus aureus remained the predominant pathogens; Pseudomonas aeruginosa and Mycoplasma pneumoniae were also increased. Combination antibiotic therapy was most frequent in patients with viral and fungal sepsis (79% and 86.5%, respectively). Patients with fungal sepsis had significantly longer hospital stays than those with viral sepsis (20.74 vs. 12.97 days, *p* = 0.017). Multivariable analysis identified that elevated lactate (OR = 1.49, 95% CI = 1.29–1.75) and pulmonary infection (OR = 2.25, 95% CI = 1.35–3.73) were independent risk factors for septic shock. Conclusions: Children with septic shock had higher mortality and prolonged hospitalization, with distinct microbiological profiles when compared with patients in the non-septic-shock group. Elevated lactate and presence of pulmonary infection are independent risk factors for septic shock. Early recognition of high-risk patients and tailored antimicrobial strategies are critical for improving outcomes.

## 1. Introduction

Sepsis remains a leading cause of morbidity and mortality in children worldwide, accounting for over 3 million deaths annually [1,2]. Survivors of pediatric sepsis and their families often face long-term consequences, including respiratory complications, reduced quality of life, recurrent severe infections, and even chronic morbidity burdens [3,4,5]. The diagnosis, pathophysiology, and management of pediatric sepsis and septic shock have long been critical areas of research. However, the diagnostic criteria for pediatric sepsis remain controversial and are under development [6,7,8,9,10,11]. In 2024, the Pediatric Sepsis Definition Task Force (PSDTF) of the international Society of Critical Care Medicine (SCCM) established the Phoenix Sepsis Criteria, which redefined pediatric sepsis as infection-associated organ dysfunction [12]. Despite this advancement, limited microbiological data and a lack of antimicrobial susceptibility profiles to guide treatment remain an important challenge in sepsis management [13]. Timely identification of pathogens could guide appropriate antibiotic therapy for this life-threatening disease. Previous studies showed that bacteria are implicated in up to 78% of pediatric sepsis cases [14,15], and severe viral illnesses may occur concurrently with bacterial infections [16,17]. Delayed initiation of antimicrobial therapy could significantly increase the risk of death in bacterial septicemia [18,19]. As sepsis could be induced by bacteria, viruses, or fungi, identifying the specific pathogens is critical for avoiding the overuse of antibiotics. Early identification of the risk factors and timely treatment could improve the prognosis of sepsis shock [20,21,22]. It is essential to develop screening tools for sepsis, identify children at high risk of sepsis, and facilitate timely interventions.

In this retrospective study, we analyzed the microbiological and clinical data of 1200 pediatric patients diagnosed with sepsis admitted to the Children’s Hospital of Chongqing Medical University between June 2018 and June 2023. All cases were classified using the Phoenix Sepsis Score criteria so as to identify pathogen distribution among pediatric sepsis patients and the risk factors associated with progression to septic shock, in order to deliver actionable insights for clinical management of pediatric sepsis.

## 2. Materials and Methods

### 2.1. Study Design and Patients

This retrospective study enrolled 1200 pediatric patients admitted to the Children’s Hospital of Chongqing Medical University (Chongqing, China) between June 2018 and June 2023 who were diagnosed with sepsis or septic shock based on the Phoenix Sepsis Score—the latest international consensus criteria. Pediatric sepsis was defined as suspected infection in children (<18 years old) accompanied by ≥2 points on the Phoenix Sepsis Score, including dysfunction in respiratory, cardiovascular, coagulation, and/or neurological systems. Septic shock was defined as sepsis with ≥1 cardiovascular point on the Phoenix Sepsis Score, including severe hypotension for age, blood lactate exceeding 5 mmol/L, or the need for vasoactive medication [12]. Clinical data were systematically extracted from electronic medical records using a standardized protocol. The data collected encompassed demographic characteristics (age, gender, medical history, etc.), hematological parameters (complete blood count, serum biochemistry, coagulation profiles, etc.), and microbiological test results (bacterial cultures, viral assays, etc.). The inclusion criteria were as follows: (1) children hospitalized with sepsis or septic shock; (2) age < 18 years; (3) complete microbiological detection data. Exclusion criteria were any of the following: (1) preterm infants and neonates hospitalized during the perinatal period; (2) Phoenix Sepsis Score ≤ 1; (3) incomplete microbiological detection data. Enrolled patients were divided into septic shock and non-septic-shock groups. The study was approved by the Ethics Committee of Children’s Hospital of Chongqing Medical University (protocol code 202577). Informed consent from the parents/guardians was waived due to the retrospective design of this study.

### 2.2. Microbiological Analysis

Microbiological data, including the bacteria, viruses, and fungi identified, were obtained from the electronic medical records of the Children’s Hospital of Chongqing Medical University. Microbiological specimens (blood, pleural/peritoneal effusions, bone marrow, etc.) obtained from hospitalized patients were processed using standardized methods: bacterial identification via blood agar cultures, viral detection via antigen-based assays or polymerase chain reaction, and fungal identification via microscopic examination.

### 2.3. Statistical Analysis

Data were analyzed using SPSS 27.0 (IBM Corp., Chicago, IL, USA) and GraphPad Prism 10.2.3 (GraphPad Software, LLC., San Diego, CA, USA) software. Continuous variables were expressed as medians with interquartile ranges (first and third quartiles); categorical variables were presented as frequencies and percentages, assessed by means of the X^2^ test. Two independent sample groups in which data did not meet the assumptions of normal distribution were assessed by the Mann–Whitney U test to compare the differences between them. Univariate and multivariate logistic regression analyses were performed to identify independent risk factors for septic shock. Variables with significant associations (*p* < 0.05) in univariate analysis were included in the multivariate model. Adjusted odds ratios (ORs) and 95% confidence intervals (CIs) were calculated for significant variables. A two-tailed *p* < 0.05 was considered statistically significant.

## 3. Results

### 3.1. Study Population

**Figure 1:**
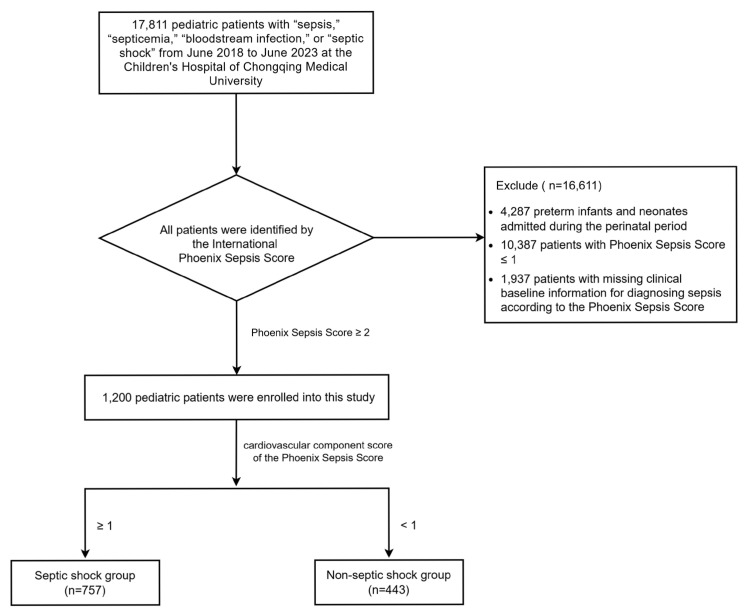
Patient enrollment flowchart.

A total of 17,811 pediatric patients were retrospectively enrolled from the Children’s Hospital of Chongqing Medical University. Among them, 16,611 patients were excluded: 4287 preterm infants and neonates admitted during the perinatal period, 10,387 patients with a Phoenix Sepsis Score ≤ 1, and 1937 patients with missing baseline clinical information for diagnosing sepsis according to the Phoenix Sepsis Score. Finally, a total of 1200 pediatric patients were enrolled in this study. Children were divided into the septic shock group (*n* = 757) and non-septic-shock group (*n* = 443) based on Phoenix Cardiovascular Score ≥ 1 (Figure 1).

### 3.2. Comparison of Baseline Characteristics in Patients with and without Septic Shock

The cohort comprised 757 septic shock patients and 443 non-septic-shock patients. The key demographic and clinical characteristics are summarized in Table 1. Significant differences were observed between groups: the age distribution differed significantly (*p* = 0.002), with septic shock patients more frequently aged < 1 month (14.9% vs. 7.4%, *p* < 0.001). Infection sources varied: the lower respiratory tract predominated in septic shock (52.2% vs. 36.9%, *p* < 0.001), while skin/wound exudates were more common in non-septic-shock patients (20.2% vs. 9.9%, *p* < 0.001). Co-detections (bacterial + viral; bacterial + fungal) were more frequent in the septic shock group, compared with the non-septic-shock group (47.1% vs. 25.0%, *p* = 0.137; 41.2% vs. 75.0%, *p* = 0.026, respectively). Pulmonary infections were significantly higher in septic shock patients (77.4% vs. 51.7%, *p* < 0.001). Clinical outcomes diverged markedly: septic shock patients required more ventilator support (51.3% vs. 26.9%, *p* < 0.001) and had longer hospital stays (14.8 vs. 12.0 days, *p* = 0.003) and higher mortality (17.4% vs. 4.7%, *p* < 0.001). Hematological parameters showed significant differences (all *p* < 0.001), including elevated lactate (2.0 vs. 1.41 mmol/L), lower PT (1.16 vs. 1.33 INR), and lower D-dimers (3.61 vs. 4.78 mg/L) in septic shock patients.

### 3.3. Epidemiological Trends of Pathogens in Pediatric Sepsis

**Figure 2:**
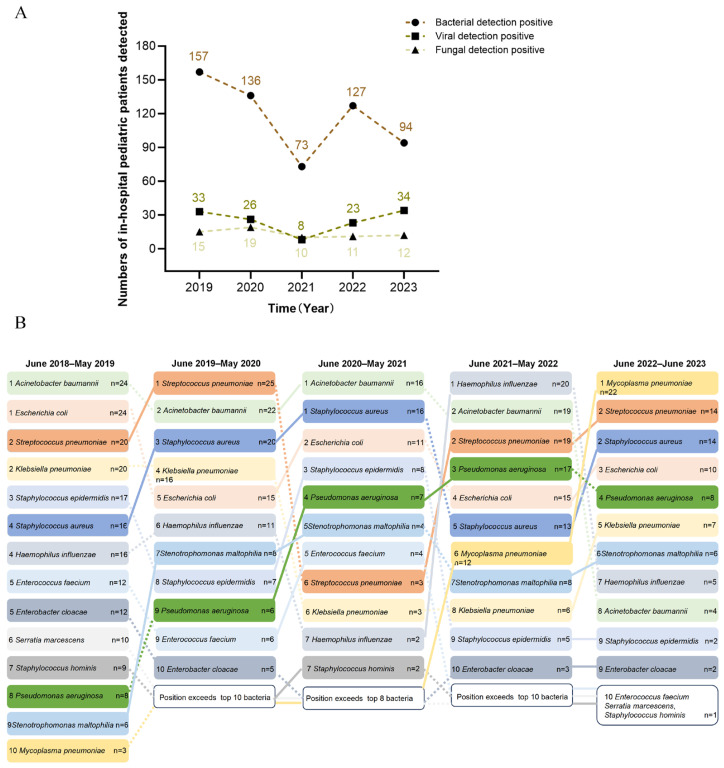
Epidemiological trends of pathogens in pediatric sepsis (2018–2023). (**A**) Annual distribution of bacterial, viral, and fungal pathogens. (**B**) Temporal changes in dominant bacterial species.

Our results indicated that bacteria predominated, followed by viruses. Positive detection rate peaks occurred in the period of June 2018–2019 (157 bacterial, 33 viral, 15 fungal cases) and troughs were seen in the period of June 2020–2021 (Figure 2A). To better understand the epidemiological trends, we comprehensively analyzed the detection results of specific bacterial pathogens over the past five years. Throughout this period, Acinetobacter baumannii (A. baumannii) remained the most prevalent bacterial pathogen, though its incidence declined after 2022. Similarly, Escherichia coli (E. coli) and Staphylococcus aureus (S. aureus) consistently ranked among the top five, while the prevalence of Streptococcus pneumoniae (S. pneumoniae) fluctuated; it initially occupied a leading position in the first two years and then experienced a significant decline from June 2020 to June 2021. Pseudomonas aeruginosa (P. aeruginosa) and Mycoplasma pneumoniae (M. pneumoniae) increased over time, whereas Klebsiella pneumoniae (K. pneumoniae) and Haemophilus influenzae (H. influenzae) declined (Figure 2B).

### 3.4. Prevalent Pathogens Between Children with and without Septic Shock

**Figure 3:**
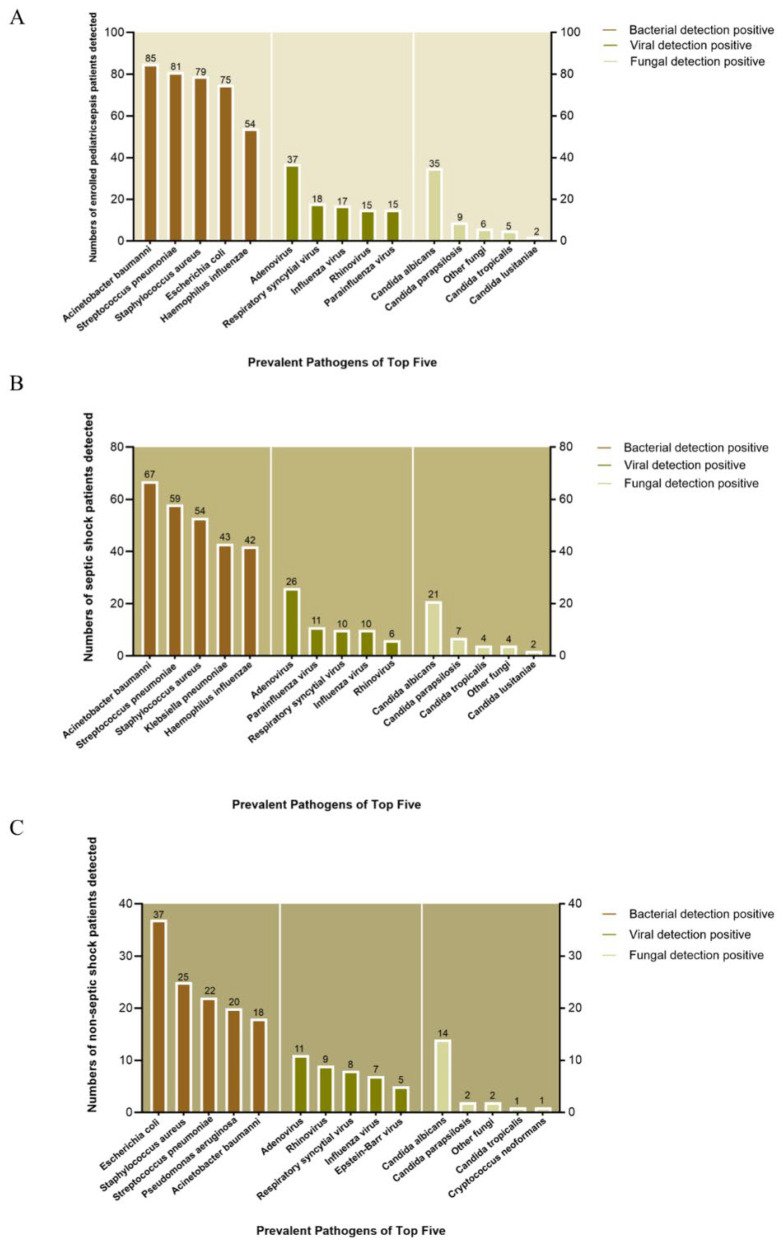
Top five pathogens in pediatric sepsis patients. (**A**) Overall cohort. (**B**) Septic shock group. (**C**) Non-septic-shock group. Pathogens are ranked by detection frequency.

We analyzed prevalent pathogens among the top five bacteria, viruses, and fungi. On the whole, A. baumannii (*n* = 85), S. pneumoniae (*n* = 81), S. aureus (*n* = 79), E. coli (*n* = 75), and H. influenzae (*n* = 54) were the top five bacteria in terms of prevalence. Adenovirus (*n* = 37), Respiratory syncytial virus (RSV) (*n* = 18), Influenza virus (*n* = 17), Rhinovirus (*n* = 15), and Parainfluenza virus (*n* = 15) ranked as the five top viruses, whereas Candida albicans (*n* = 35), Candida parapsilosis (*n* = 9), other fungi (*n* = 6), Candida tropicalis (*n* = 5), and Candida lusitaniae (*n* = 2) were the top five most prevalent fungal pathogens (Figure 3A).

However, the bacterial distribution varied between groups. In patients with septic shock, A. baumannii (*n* = 67), S. pneumoniae (*n* = 59), S. aureus (*n* = 54), K. pneumoniae (*n* = 43), and H. influenzae (*n* = 42) predominated, while non-septic-shock patients showed higher E. coli (*n* = 37), S. aureus (*n* = 25), S. pneumoniae (*n* = 22), P. aeruginosa (*n* = 20), and A. baumannii (*n* = 18) prevalence (Figure 3B,C).

Viruses also differed between the groups. Adenovirus (*n* = 26), Parainfluenza virus (*n* = 11), RSV (*n* = 10), Influenza virus (*n* = 10), and Rhinovirus (*n* = 6) were more prevalent in the septic shock group, compared with Adenovirus (*n* = 11), Rhinovirus (*n* = 9), RSV (*n* = 8), Influenza virus (*n* = 7), and Epstein–Barr virus (*n* = 5) in the non-septic-shock group.

However, the distribution of fungi was similar in the two groups. Candida albicans (*n* = 21), Candida parapsilosis (*n* = 7), Candida tropicalis(*n* = 4), other fungi (*n* = 4), and Candida lusitaniae (*n* = 2) were the top five in the septic shock group, while Candida albicans (*n* = 14), Candida parapsilosis (*n* = 2), other fungi (*n* = 2), Candida tropicalis (*n* = 1), and Cryptococcus neoformans (*n* = 1), in that order, ranked in the top five in the non-septic-shock group.

### 3.5. Prevalent Pathogens in Non-Survivors with and without Septic Shock

**Figure 4:**
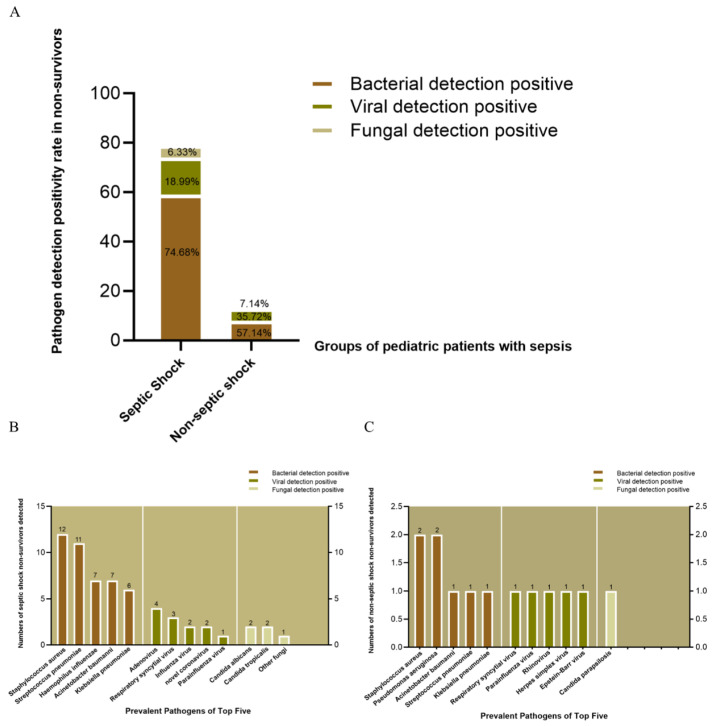
Pathogen distribution in non-survivors. (**A**) Positivity rates of bacterial, viral, and fungal pathogens (no significant intergroup differences, *p* > 0.05). (**B**) Top pathogens in septic shock non-survivors. (**C**) Top pathogens in non-septic-shock non-survivors.

Given that sepsis is a leading cause of death among hospitalized children and is characterized by high incidence and mortality, we analyzed the distribution of pathogens in non-survivors with and without septic shock whose deaths were defined as in-hospital deaths attributed to sepsis or septic shock. In children with septic shock, the detection rates of bacterial, viral, and fungal pathogens among non-survivors were 74.68%, 18.99%, and 6.33%, respectively. In contrast, in the non-septic-shock group, the detection rates were 57.14% for bacteria, 35.72% for viruses, and 7.14% for fungi (*p* > 0.05 for all comparisons) (Figure 4A).

We further identified the top five pathogens in both groups. In the septic shock group, the top five bacterial pathogens were S. aureus (*n* = 12), S. pneumoniae (*n* = 11), H. influenzae (*n* = 7), A. baumannii (*n* = 7), and K. pneumoniae (*n* = 6). In contrast, the non-septic-shock group showed lower prevalence, with S. aureus (*n* = 2), P. aeruginosa (*n* = 2), A. baumannii (*n* = 1), S. pneumoniae (*n* = 1), and K. pneumoniae (*n* = 1) as the top pathogens.

In terms of viral pathogens, the septic shock group exhibited higher positivity for Adenovirus (*n* = 4), Respiratory syncytial virus (*n* = 3), Influenza virus (*n* = 2), SARS-CoV-2 (*n* = 2), and Parainfluenza virus (*n* = 1). The non-septic-shock group had RSV (*n* = 1), Parainfluenza virus (*n* = 1), Rhinovirus (*n* = 1), Herpes simplex virus (*n* = 1), and Epstein–Barr virus (*n* = 1).

Fungal pathogens in the septic shock group included Candida albicans (*n* = 2), Candida tropicalis (*n* = 2), and other fungi (*n* = 1), while only Candida parapsilosis (*n* = 1) was detected in the non-septic-shock group (Figure 4B,C).

### 3.6. Antibiotic Utilization in Viral and Fungal Infection Between Patients in the Septic Shock and Non-Septic-Shock Groups

Analysis of antibiotic utilization patterns in pediatric bacterial sepsis revealed combination therapy as the predominant approach. β-Lactam antibiotics comprised 48% (265/552) of all regimens administered. Within this cohort, the observed in-hospital mortality rate was 11.6% (64/552).

Aiming to evaluate the rational use of antimicrobial agents in clinical practice, we compared antibiotic administration in viral and fungal sepsis among all children with sepsis enrolled. Among the 100 viral sepsis patients, combination antibiotic therapy predominated (79%, 79/100), and 21% received monotherapy. As for fungal sepsis, 86.5% (32/37) received combination therapy and 13.5% (5/37) monotherapy with a non-fungal antibiotic. A comparison of the antibiotic medication regimens between viral sepsis and fungal sepsis patients demonstrated a significant difference (*p* = 0.023).

In viral sepsis, antibiotic regimens included β-lactam monotherapy (67%, 67/100), β-lactam + macrolide (4%, 4/100), β-lactam + quinolone (1%, 1/100), β-lactam + vancomycin (20%, 20/100), and other combinations (8%, 8/100). For fungal sepsis, regimens were distributed as follows: β-lactam monotherapy (43.2%, 16/37), β-lactam + macrolide (8.1%, 3/37), β-lactam + vancomycin (18.9%, 7/37), and other combinations (29.7%, 11/37). Ventilator support was required in 37% (37/100) of viral sepsis and 45.9% (17/37) of fungal sepsis patients.

Hospital stays differed significantly between the viral and fungal sepsis groups (12.97 vs. 20.74 days, *p* = 0.017). However, no statistically significant difference was found in in-hospital mortality (16% vs. 8.1%, *p* = 0.235) in the two groups (Table 2).

### 3.7. Univariate and Multivariate Analysis of Risk Factors Associated with Septic Shock

Univariate analysis identified bacterial positivity (OR: 1.426; 95% CI: 1.127–1.807; *p* = 0.003), age < 1 month (OR: 2.180; 95% CI: 1.467–3.321; *p* < 0.001), pulmonary infection (OR: 3.000; 95% CI: 2.171–4.156; *p* < 0.001), and elevated lactate (OR: 1.408; 95% CI: 1.286–1.557; *p* < 0.001) as significant risk factors for septic shock. The platelet count (PLT), platelet-to-mean platelet volume ratio (PLT/MPV), and red cell distribution width (RDW) were also associated with septic shock (*p* < 0.001 for all). Viral and fungal positivity showed no significant association. Multivariate analysis confirmed elevated lactate (OR: 1.487; 95% CI: 1.294–1.751; *p* < 0.001) and pulmonary infection (OR: 2.246; 95% CI: 1.351–3.734; *p* = 0.002) as independent risk factors (Table 3).

## 4. Discussion

Sepsis is a life-threatening disease response to infection, associated with critical morbidity and mortality in children [23]. Pediatric-specific sepsis criteria were first published in 2005 based on expert opinion and were widely applied in clinical practice and research [6]. In 2016, the Third International Consensus Definitions for Sepsis and Septic Shock (Sepsis-3) revised the criteria for adults but excluded pediatric populations [10]. By 2024, the Phoenix Sepsis Score criteria for pediatric sepsis and septic shock were derived and published [12]. However, few studies have investigated the microbiological and clinical characteristics of septic shock and non-septic shock in pediatric patients using the Phoenix Sepsis Score.

In our cohort of 1200 patients, septic shock patients exhibited significantly higher in-hospital mortality than non-septic-shock patients (17.4% vs. 4.7%, *p* < 0.001). Our results are aligned with the study of Schlapbach et al., who showed that the in-hospital mortality of children with a Phoenix score of ≥2 was 7.1% in developed and 28.5% in developing areas [12]. Previous studies indicated that the prevalence of bacteremia in hospitalized children, determined via blood cultures, was 2.2% (46/2143) [24], which was remarkably lower than that in this cohort of children. One possible reason for this is that our study detected pathogens from all specimen types (blood, lower respiratory tract secretions, skin/wound exudates, catheter and pleural/abdominal effusion, cerebrospinal fluid, bone marrow feces, urine, etc.). In this five-year analysis, we found bacterial infection (48.92%, 587/1200) was the most prevalent among sepsis patients, followed by viral (10.3%, 124/1200), which is consistent with the report of the Sepsis Prevalence, Outcomes, and Therapies (SPROUT) study (bacterial source in 65.4% and viral source in 20.9% of 567 children with severe sepsis) [15]. In addition, our results showed that single bacterial infections predominated in both the septic and non-septic-shock groups, with fewer cases of dual or polybacterial infections. Our results are consistent with those of Salud et al., who categorized pathogens prospectively in children with septic shock and indicated that single pathogens predominated (single bacterial/fungal, *n* = 125; single viral, *n* = 91) over mixed infections (*n* = 70) [25]. We identified four co-detection patterns in the septic shock group: bacterial–viral (*n* = 16), bacterial–fungal (*n* = 14), viral–fungal (*n* = 1), and multi-pathogen (*n* = 3). The non-septic-shock group showed only bacterial–viral (*n* = 4) and bacterial–fungal (*n* = 12) co-detections.

The prevalence of bloodstream infection pathogens varies with age, comorbidities, and socioeconomic factors. Common pathogens in children >1 month include S. pneumoniae, S. aureus, Neisseria meningitidis, and E. coli [26]. Another study identified H. influenzae and K. pneumoniae as the predominant Gram-negative bacteria, while S. aureus and S. pneumoniae were the most common Gram-positive pathogens [27]. In our cohort, A. baumannii, S. pneumoniae, S. aureus, E. coli, and H. influenzae ranked in the top five bacteria in terms of prevalence. Among non-survivors, S. aureus, S. pneumoniae, H. influenza, A. baumannii, and K. pneumoniae were the top five bacteria in the septic shock group, while S. aureus, P. aeruginosa, A. baumannii, S. pneumoniae, and K. pneumoniae were prevalent in the non-septic-shock group.

Viral pathogens such as Rhinovirus respiratory syncytial virus (RSV) and Adenovirus were frequently detected in the SPROUT study [15]. Similarly, RSV, Cytomegalovirus, Epstein–Barr virus, Herpes simplex virus, Varicella-zoster virus, and Influenza were common in Australian/New Zealand pediatric sepsis cohorts [28]. Our results are aligned with these findings, with Adenovirus, Parainfluenza virus, RSV, Influenza virus, and Rhinovirus predominant in the septic shock group and Adenovirus, Rhinovirus, RSV, Influenza virus, and Epstein–Barr virus in the non-septic-shock group.

The early differentiation of specific bacterial, viral, and fungal pathogens in sepsis patients remains challenging in clinical practice. This results in routine antibiotic use, even in suspected viral or fungal cases [29]. In our study, combination therapy was predominant both in viral (79%) and fungal (86.5%) sepsis patients. β-lactam antibiotics were most frequently used. Fungal sepsis patients received more β-lactam, macrolide, and vancomycin combination therapies. Fungal sepsis patients also had significantly longer hospital stays than viral sepsis patients (20.74 vs. 12.97 days, *p* = 0.017). To address this, antibiotic stewardship must prioritize rapid pathogen differentiation within 24 h of admission via multiplex PCR and biomarker testing (e.g., procalcitonin) to enable early de-escalation. Specifically for viral sepsis, β-lactam monotherapy should be restricted to confirmed bacterial comorbidities, with antibiotics discontinued within 48–72 h upon viral confirmation; conversely, for fungal sepsis, empirical antifungals require strict risk stratification, while infectious disease consultations become mandatory for complex regimens. The aim of these evidence-based strategies is decreasing antibiotic exposure, thereby reducing the risk of antimicrobial resistance and shortening hospital stays, directly countering the suboptimal outcomes observed.

Multivariate analysis identified elevated lactate (OR 1.487, 95% CI 1.294–1.751; *p* < 0.001) and pulmonary infection (OR 2.246, 95% CI 1.351–3.734; *p* = 0.002) as independent risk factors for septic shock. Kamran et al. evaluate the prognostic value of the lactate-to-albumin (L/A) ratio compared to that of lactate and lactate clearance in predicting the outcomes in patients with septic shock [30]. Heemoon et al. indicated that elevated lactate (≥4 mmol/L) is a key indicator for progression to septic shock, necessitating early resuscitation to improve outcomes [31]. He et. showed that pulmonary infection is a risk predictor linked to increased mortality and reduced quality of life [32].

Nevertheless, there are some limitations in this study: its single-center retrospective design constrains the generalizability of the findings, particularly given regional variations in pathogen epidemiology and healthcare resource allocation; a potential selection bias arises from excluding preterm neonates and perinatal hospitalizations, thereby underrepresenting high-risk populations; and retrospective data collection resulted in the exclusion of cases with incomplete Phoenix Score parameters, potentially compromising outcome validity. Consequently, future multicenter prospective cohorts should validate pathogen distributions and shock risk factors across diverse socioeconomic settings while incorporating comorbidity burden and antimicrobial resistance patterns into predictive models, quantify economic and resistance impacts of stewardship interventions, and establish evidence-based timelines for antibiotic de-escalation—collectively addressing these constraints while advancing clinical translation.

In summary, our study highlights distinct microbiological and clinical profiles in pediatric septic shock and non-septic-shock patients. Septic shock patients exhibited higher mortality and prolonged hospital stays. Hyperlactatemia and pulmonary infection were independent risk factors for septic shock. These findings underscore the need for optimized diagnostic and therapeutic strategies, as well as larger prospective studies to validate and expand upon these results.

## Figures and Tables

**Table 1:** Baseline clinical characteristics of pediatric sepsis patients with and without septic shock.

Characteristics		Enrolled Children with Sepsis	Septic Shock Group (*n* = 757)	Non-Septic-Shock Group (*n* = 443)	Z/(X^2^)	*p* Value
Gender (*n*,%)					(1.972)	0.160
	Male	670(55.8%)	411(54.3%)	259(58.5%)		
	Female	530(44.2%)	346(45.7%)	184(41.5%)		
Age (*n*,%)					(18.792)	0.002
	<1 month	146(12.2%)	113(14.9%)	33(7.4%)	(14.624)	<0.001
	1–11 months	305(25.4%)	182(24.0%)	123(27.8%)		
	1–2 years	152(12.7%)	90(11.9%)	62(14.0%)		
	2–5 years	244(20.3%)	147(19.4%)	97(21.9%)		
	5–12 years	255(21.2%)	169(22.4%)	86(19.4%)		
	12–18 years	98(8.2%)	56(7.4%)	42(9.5%)		
Site of pathogen detection (*n*,%)					(39.685)	<0.001
	Lower respiratory tract	371(47.3%)	278(52.2%)	93(36.9%)	(15.971)	<0.001
	Blood	204(26.0%)	141(26.5%)	63(25.0%)	(0.188)	0.665
	Skin/wound discharge	104(13.2%)	53(9.9%)	51(20.2%)	(15.777)	<0.001
	Catheter	37(4.7%)	27(5.1%)	10(4.0%)	(0.459)	0.498
	Pleural/abdominal effusion	22(2.8%)	14(2.6%)	8(3.2%)	(0.189)	0.664
	Cerebrospinal fluid	10(1.3%)	2(0.4%)	8(3.2%)	(10.661)	0.001
	Other	37(4.7%)	18(3.3%)	19(7.5%)	(6.601)	0.010
Types of bacterial detection					(9.578)	0.023
	No bacteria detection	613(51.1%)	362(47.8%)	251(56.7%)	(8.737)	0.003
	Single bacterial detection positive	442(36.8%)	294(38.8%)	148(33.4%)	(3.540)	0.060
	Dual bacterial detection positive	100(8.3%)	68(9.0%)	32(7.2%)	(1.132)	0.287
	Polybacterial detection positive	45(3.8%)	33(4.4%)	12(2.7%)	(2.109)	0.146
Types of viral detection					(2.250)	0.522
	No virus detected	1076(89.7%)	683(90.2%)	393(88.7%)	(0.689)	0.407
	Single viral detection positive	111(9.2%)	67(8.9%)	44(10.0%)	(0.389)	0.533
	Dual viral detection positive	12(1.0%)	7(0.9%)	5(1.1%)	(0.117)	0.732
	Polyviral detection positive	1(0.1%)	0	1(0.2%)	(1.710)	0.191
Types of fungal detection					(1.521)	0.217
	No fungus detected	1133(94.4%)	710(93.8%)	423(95.5%)		
	Fungal detection positive	67(5.6%)	47(6.2%)	20(4.5%)		
Types of pathogen co-detection					(5.600)	0.133
	Bacterial plus viral detection positive	20(40.0%)	16(47.1%)	4(25.0%)	(2.206)	0.137
	Bacterial plus fungal detection positive	26(52.0%)	14(41.2%)	12(75.0%)	(4.987)	0.026
	Viral plus fungal detection positive	1(2.0%)	1(2.9%)	0	(0.480)	0.488
	Multi-pathogen detection positive	3(6.0%)	3(8.8%)	0	(1.502)	0.220
Pulmonary infection (*n*,%)					(85.243)	<0.001
	Yes	815(67.9%)	586(77.4%)	229(51.7%)		
	No	189(15.8%)	87(11.5%)	102(23.0%)		
	Unknown	196(16.3%)	84(11.1%)	112(25.3%)		
Ventilator support (*n*,%)					(68.149)	<0.001
	Yes	507(42.3%)	388(51.3%)	119(26.9%)		
	No	693(57.7%)	369(48.7%)	324(73.1%)		
WBC[×10^9^/L,*M*(*P*25,*P*75)]		11.88(6.64,18.02)	11.8(6.68,17.82)	12.22(6.46,18.24)	–0.010	0.992
PLT[×10^9^/L,*M*(*P*25,*P*75)]		226(108.5,371)	254(143,392.5)	163(68.75,311.25)	7.066	<0.001
PLT/MPV[×10^9^/(L×fL),*M*(*P*25,*P*75)]		25.09(14.17,38.72)	26.57(16.05,40.02)	22.79(9.34,34.93)	4.053	<0.001
NLR/*M*(*P*25,*P*75)		2.81(1.36,6.16)	2.59(1.35,5.45)	3.38(1.39,7.56)	–2.839	0.005
PDW/*M*(*P*25,*P*75)		12.1(10.5,14.6)	12(10.5,14.4)	12.2(10.6,15.1)	–1.213	0.225
RDW/*M*(*P*25,*P*75)		14.9(13.5,19.8)	15.1(13.6,26.5)	14.5(13.4,17.38)	3.186	0.001
PT/*M*(*P*25,*P*75)		1.17(1.06,1.43)	1.16(1.04,1.38)	1.33(1.12,1.47)	–6.041	<0.001
D-dimer/*M*(*P*25,*P*75)		4.03(2.17,9.70)	3.61(1.82,9.21)	4.78(2.62,11.08)	–4.963	<0.001
Lactic Acid/[mmol/L,*M*(*P*25,*P*75)]		1.8(1.1,3.58)	2(1.2,4.4)	1.41(0.9,2.4)	6.797	<0.001
In-hospital stay/[d,M(P25,P75)]		13.65(7.59,23.76)	14.8(7.6,27.3)	12.0(7.6,19.4)	2.982	0.003
In-hospital mortality (*n*,%)					–40.498	<0.001
	Yes	153(12.8%)	132(17.4%)	21(4.7%)		
	No	1047(87.2%)	625(82.6%)	422(95.3%)		

Data are presented as medians (interquartile ranges, IQRs) or frequencies (%). Abbreviations: WBC, white blood cell; PLT, platelet count; PLT/MPV, platelet/mean platelet volume; NLR, neutrophil-to-lymphocyte ratio; PDW, platelet distribution width; RDW, red cell distribution width; PT, prothrombin time. Continuous variables were compared using the Mann–Whitney U test; categorical variables were assessed with X^2^ tests. *p* values < 0.05 were considered statistically significant.

**Table 2:** Antibiotic utilization and clinical outcomes in viral vs. fungal sepsis.

Characteristics	Bacterial Sepsis Patients (*n* = 552)	Viral Sepsis Patients (*n* = 100)	Fungal Sepsis Patients (*n* = 37)	*p* Value
Types of antibiotic medication (*n*,%)				0.321
		
Monotherapy	38(6.8%)	21(21%)	5(13.5%)	
Combination therapy	514(93.2%)	79(79%)	32(86.5%)	
Antibiotic medication regimen (*n*,%)				0.023
		
β-lactam antibiotics	265(48%)	67(67%)	16(43.2%)	
β-lactam antibiotics plus macrolide antibiotics	47(8.5%)	4(4%)	3(8.1%)	
β-lactam antibiotics plus quinolone antibiotics	41(7.4%)	1(1%)	1(2.7%)	
β-lactam antibiotics plus vancomycin	152(27.5%)	20(20%)	7(18.9%)	
β-lactam antibiotics plus macrolide antibiotics plus other antibiotics	1(0.2%)	1(1%)	4(10.8%)	
β-lactam antibiotics plus vancomycin plus other antibiotics	46(8.4%)	6(6%)	6(16.3%)	
Only vancomycin	0	1(1%)	0	
Ventilator support (*n*,%)				0.341
Yes	274(49.6%)	37(37%)	17(45.9%)	
No	278(50.4%)	63(63%)	20(54.1%)	
In-hospital stay/[d,M(*P*25,*P*75)]	16.07(9.40,29.94)	12.97(6.38,19.52)	20.74(11.37,29.46)	0.017
In-hospital mortality (*n*,%)				0.235
Yes	64(11.6%)	16(16%)	3(8.1%)	
No	488(88.4%)	84(84%)	34(91.9%)	

Data are expressed as frequencies (%) or medians (IQRs). Between-group differences were analyzed using X^2^ tests (categorical variables) and Mann–Whitney U tests (continuous variables). *p* values < 0.05 were considered statistically significant.

**Table 3:** Univariate and multivariate logistic regression analysis of risk factors for septic shock.

Variables	Univariate			Multivariate		
	OR	95%CI	*p* Value	OR	95%CI	*p* Value
Bacterial detection positive	1.426	1.127–1.807	0.003	1.152	0.771–1.724	0.489
Viral detection positive	0.852	0.584–1.250	0.407			
Fungal detection positive	1.400	0.830–2.445	0.219			
Age (<1month, ≥1month)	2.180	1.467–3.321	<**0.001**	2.062	1.001–4.498	0.058
PLT	1.002	1.001–1.003	<**0.001**	0.999	0.992–1.006	0.824
PLT/MPV	1.015	1.007–1.023	<**0.001**	1.019	0.953–1.089	0.588
PT	0.936	0.802–1.095	0.399			
D-dimer	0.998	0.989–1.007	0.594			
NLR	0.984	0.968–1.001	0.062			
RDW	1.016	1.007–1.026	<**0.001**	1.01	0.993–1.029	0.249
Lactic acid	1.408	1.286–1.557	<**0.001**	1.487	1.294–1.751	<**0.001**
Pulmonary infection	3.000	2.171–4.156	<**0.001**	2.246	1.351–3.734	**0.002**

Abbreviations: OR, odds ratio; CI, confidence interval; PLT, platelet count; MPV, mean platelet volume; NLR, neutrophil-to-lymphocyte ratio; RDW, red cell distribution width; PT, prothrombin time. Variables with *p* < 0.05 in univariate analysis were included in the multivariate model. Significant results (*p* < 0.05) are highlighted in bold.

## Data Availability

The data presented in this study are available upon request from the corresponding author. The data are not publicly available due to ethical and privacy restrictions.

## Abbreviations

The following abbreviations are used in this manuscript:

OR	Odds ratio
CI	Confidence interval
A. baumannii	Acinetobacter baumannii
E. coli	Escherichia coli
S. aureus	Staphylococcus aureus
S. pneumoniae	Streptococcus pneumoniae
P. aeruginosa	Pseudomonas aeruginosa
M. pneumoniae	Mycoplasma pneumoniae
K. pneumoniae	Klebsiella pneumoniae
H. influenzae	Haemophilus influenzae
NLR	Neutrophil-to-lymphocyte ratio
RSV	Respiratory syncytial virus
PLT	Platelet count
PLT/MPV	Platelet-to-mean platelet volume ratio
RDW	Red cell distribution width
L/A	Lactate-to-albumin ratio
